# Influence of Enteral Nutrition on Gut Microbiota Composition in Patients with Crohn’s Disease: A Systematic Review

**DOI:** 10.3390/nu12092551

**Published:** 2020-08-23

**Authors:** Paulina Horwat, Stanisław Kopeć, Aleksandra Garczyk, Iwona Kaliciak, Zuzanna Staręga, Konstanty Drogowski, Marcin Mardas, Marta Stelmach-Mardas

**Affiliations:** 1 Department of Biophysics, Poznan University of Medical Science, 60-780 Poznan, Poland; paulina.horwat9@gmail.com (P.H.); s.kopec@macron.pl (S.K.); garczykaleksandra@gmail.com (A.G.); ikaliciak@o2.pl (I.K.); zuziastarega@wp.pl (Z.S.); drogowskos@wp.pl (K.D.); 2 Department of Oncology, Poznan University of Medical Science, 60-569 Poznan, Poland; marcin.mardas@ump.edu.pl

**Keywords:** inflammatory bowel disease, nutrition, time of remission, malnutrition

## Abstract

The aim of the study was to systematically and comprehensively evaluate whether exclusive enteral nutrition (EEN) has impact on gut microbiota in patients with Crohn’s disease (CD). The databases PUBMED (MEDLINE), SCOPUS and WEB OF SCIENCE were searched. Out of 232 studies, 9 met inclusion criteria. The combined analyzed population consists of 118 patients with CD and treated with EEN with a time of intervention of 2–12 weeks. Studies were conducted in children, with the exception of one study. All applied feeding formulas had similar energy value and composition. The microbiome analysis was based on 16S rRNA gene sequencing of faecal samples. In all studies, EEN treatment decreases inflammatory markers (i.e., hs-CRP and FCP). A change in abundance of numerous bacterial families (*Clostridiaceae, Eubacteriaceae, Bacteroidaceae*) was noticed, especially in *Bacteroidaceae*. An increase in families connected to the more severe clinical course (*Fusobacteria, Prevotella, Lachnospiraceae*) was observed in only 2.5% of CD patients. Our analyses suggest EEN has a beneficial influence on gut microbiome in patients with CD, which is interrelated with clinical patient’s improvement and time of disease remission.

## 1. Introduction

Inflammatory bowel disease (IBD) comprises Crohn’s disease (CD) and Ulcerative colitis (UC) [1]. Interactions between genes and environmental factors are associated with the pathogenesis of IBD, where the prevalence is the highest in “Western countries” characterized by diets rich in fat and protein [1,2,3]. CD is connected to the damage of the mucosa in the gastrointestinal tract (GI) and followed by the impaired absorption of nutrients, which results in malnutrition in 20–85% of patients [4,5]. The damage of the mucosal layer, aggravated retardation of nutrients and direct interactions between prescribed drugs and selected food components, influence the restrictions of administered food in this group of patients [6,7]. An Exclusive enteral nutrition (EEN) regimen is the first-line therapy in CD for pediatric patient period. One recommendation of the European Society for Clinical Nutrition and Metabolism (ESPEN) guideline [7] indicates that EEN should always be preferred over the parenteral route. From a clinical point of view, an introduction of EEN in CD patients may bring high remission rate as the results of the microbiota changes composition related to the reduction of proinflammatory microbial components and harmful microbial metabolites [8,9]. It is worth mentioning that for the last few years, the strong association between different bacteria (i.e., *E. coli, Bacteroides fragilis*) and inflammation of the mucosal lining of the GI tract has been noticed. Gut dysbiosis is thought to be the result of inflammation and can be triggered by various external factors [10]. The type of diet, including EEN, seems to play an important role not only in achieving but also in remission maintenance in IBD patients [6,10].

Therefore, the aim of this study was to evaluate the changes of gut microbiota composition during EEN treatment.

## 2. Experimental Section

### 2.1. Search Strategy, Inclusion and Exclusion Criteria

From November 2019 to May 2020 the research of the following databases PUBMED (MEDLINE), SCOPUS and WEB OF SCIENCE was processed in order to identify the experimental and observational studies that investigate a change in the gut microbiome after the exclusive or partial enteral nutrition (PEN) therapy among patients with CD. The search strategy was restrained to the human population and the English language. Original articles were included. No restrictions regarding the date of the publication or age of patients were used. In all cases, the diagnosis was made using inflammatory markers, endoscopic findings, and/or symptoms. Taking into account study design, the following articles were included: case studies, randomized controlled trials, nonrandomized controlled trials and cohort studies, where the EEN or PEN was used as a treatment method. The articles with low quality data or incomplete data that could not be fully obtained from authors were excluded. Moreover, patients with administered probiotics or not fully diagnosed were also ineligible for this review.

The search strategy included the following index terms: 1# Inflammatory bowel disease OR Crohn disease; 2# Microbiota OR Human Microbiome OR Microbial Community OR Microbial Community Composition OR Microbial Community Structure OR Metagenome; 3# Enteral nutrition OR Enteral Feeding OR Force Feeding OR Gastric Feeding Tubes OR Tube Feeding; #4 #1 AND #2 AND #3.

### 2.2. Data Extraction and Analysis

In the first stage of the study selection, the titles of the articles were initially reviewed by three different teams, each one contained two researchers. Every team searched one of the databases. All records selected in the title review phase were further reviewed by the abstracts and assessed for eligibility. Afterward, teams presented their search outcomes to each other and the decision on the article inclusion was made collaboratively. Limited data or no possible contact with authors excluded studies during the full text assessment stage. From each qualified study, the following data were extracted: title, main author, publication year, study name and design, countries involved and the total number of patients. Regarding the population characteristic, the following information about patients was derived: age, sex, ethnicity, Body Mass Index (BMI), diagnosis, disease location, medications, duration of the disease, remission during treatment, high sensitivity C-reactive protein (hs-CRP), and faecal calprotectin (FCP) levels, Crohn’s Disease Activity Index (CDAI) or Pediatric Crohn’s Disease Activity Index (PCDAI). Briefly, the CDAI is defined on the basis of the intensity of the 8 symptoms presented by the patient (number of liquid stools, abdominal pain, general well-being, arthralgia, mucocutaneous lesions, iritis, anal disease, external fistula, fever, use of antidiarrheal, abdominal mass, hematocrit and body weight). Furthermore, it is applied in adult patients and the value above 150 indicates an active disease [11]. In pediatric patients, the PCDAI is used, which ranges between 0 and 100. Scores lower than 10 are indicative of the inactive disease while above 30 indicates severe disease. Additionally, the nutritional composition of provided enteral nutrition products was obtained. The microbiome structure was assessed with the use of the traditional microbiological techniques, metagenome sequencing, 16sRNA analysis and PCR. In order to describe the microbiome in the quantitative manner, the Shannon Diversity Index, abundance of bacteria and bacterial diversity were used.

### 2.3. Statistical Approach

The taxon of bacteria family was used in the data presentation according to the NCBI Taxonomy Browser system. In order to compare the changes in each of the studies, the percentage of changes was calculated, according to the following formula: [difference/initial quantity × 100%]. The quantitative changes have been shown as increased or decreased and expressed as percentage. Taking under consideration the heterogeneity of the data, quantities were transformed into one unit: mean or an operational taxonomic unit (OTU’s). The model of linear regression was used to calculate the number of each bacteria family (following the formula y = ax + b).

## 3. Results

### 3.1. Search Results

**Figure 1:**
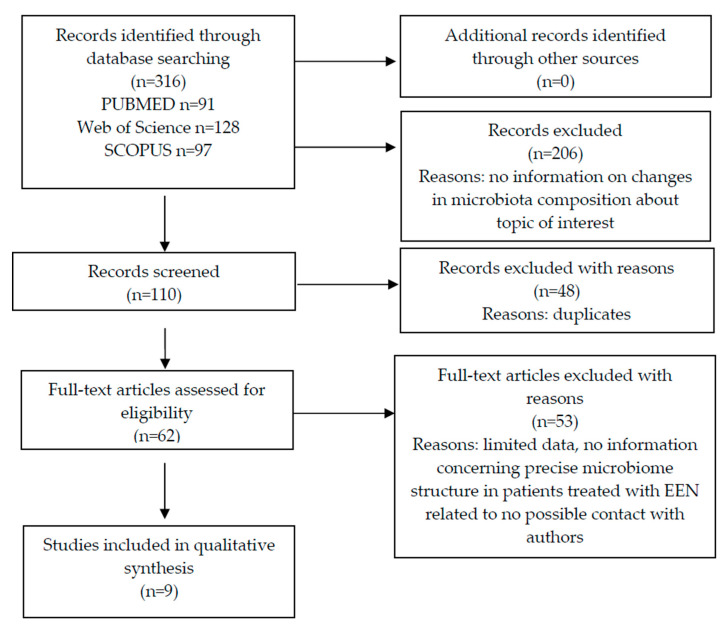
Flow chart of the databases search on the changes in the gut microbiota during exclusive enteral nutrition treatment.

The flow chart of studies search is presented in Figure 1. Based on the title database search, 316 articles were extracted. After the review of the abstracts, 206 studies were excluded and 25 positions were carefully examined. Because of the limited data or no possible contact with authors, 11 of them were removed. From the 62 full-text articles, 53 were excluded because of the lack of precise data or given information did not concern microbiome structure (e.g., bacterial metabolites) or gene pathways. During precise interpretation of the results and information presented in the studies, 9 articles met the inclusion and exclusion criteria in order to answer the primary research question.

### 3.2. Characteristics of the Included Studies and Study Population

The characteristics of included studies are presented in Table 1. Most of the studies were nonrandomized clinically controlled trials conducted in European population (80%). The total number of patients diagnosed with CD and treated with EEN was 118, with the time of intervention ranged between 2 and 12 weeks. Only one article [12] considered feeding with PEN and 9 patients from study conducted by Shiga et al. [10] had total parenteral nutrition (TPN) administered. Most of the studies [4,8,12,13,14,15,16,17] were conducted among children. All applied feeding formulas had similar energy value and composition, with exception of one study [10], characterized by greater percentage of carbohydrates. One of the studies [13] treated patients (mean age of patients: 8.5) with an amino-acid formula intended for infants.

The detailed clinical characteristic of study patients is presented in Table 2. All patients were diagnosed with active CD with mean CDAI > 65. Almost 70% of patients were newly diagnosed or with history of disease shorter than 1.5 years. The only exception was the study preformed in an adult population [10], where the disease duration was up to 22 years. At least 72% of subjects had involvement of the upper digestive tract (L3), ileocolon (L4) or upper digestive tract and ileocolon (L3 + L4). Furthermore, 8% of the study population underwent antibiotic therapy and 12% underwent steroid therapy. The percentage of patients receiving “other” additional medications in individual studies varied widely. The mean time of disease remission varied between studies (2.5–26 weeks) and was associated with the duration of provided EEN and based on the value of PCDAI (<15) or CDAI (<150). Enhancement of the patient state can be identified if the value drops down at least 12.5 points [18] which corresponds with decreased PCDAI/CDAI values during the EEN treatment and changes in C-reactive protein (CRP) and faecal calprotectin (FCP) concentrations.

### 3.3. Microbiome Changes during EEN

The microbiome analysis was based on faecal samples, with exception of one study [14] where sample of ileum tissue was used. The bacteria categorization (i.e., OTU’s) that based on similarity in microbiota composition over the treatment period was used in order to comprise research about their changes (Table 3). Only two studies [4,14] included comparison of the changes in Shannon Diversity Index of CD patients during EEN. The obtained results were subtracted from the quantity before EEN treatment from the residual amount and described each family as beneficial or pathogenic. The obtained microbiota structure was compared to the architecture broadly considered as typical for the healthy subjects, where the dominance of the *Bacteroides* and *Firmicutes* family and the limited amount of the *Proteobacteria* is observed (Table 4). Presence of the *Erysipelotrichaceae, Ruminococcaceae, Lachnospiraceae, Streptococcaceae, Veillonellaceae, and Peptostreptococcaceae* families was linked to the enhancement of the inflammation [13]. *Ruminococcae* prevalence was reduced in five [2,9,13,16,17] out of 6 studies [2,9,12,13,16,17]. *Lachnospiraceae* abundance was intensified in all of the studies, expect of one [13]. *Enterococcaceae,* which is associated with many healthcare-acquired inflammations, especially among people with immunosuppression, showed a 70% decrease [12]. A decline in *Veillonellaceae* among all study patients was observed. Furthermore, the decrease alone was seen in *Enterobacteriaceae* being commonly associated with intestinal mucosa degradation and inflammation. The data regarding the *Bacteroidaceae* changes over EEN treatment are inconsistent, where marked increase was observed in 3 studies [4,13,14], while a decrease was visible that could indicate the disease progression or be due to the outcome of the reduced diversity after EEN therapy in another 2 studies [12,15]. Although *Bifidobacteriaceae* is considered as a valuable family in the GI tract, a decrease in their abundance was indicated in 3 studies [4,15,16], with reductions up to 42.79%, 99.82% and 60.19%, respectively, observed. Finally, the reduction of the *Prevotellaceae* family was noted [4,12,13,15], where in the case of one study [12] fully retired. An abundance of *Coriobacteriaceae* was enhanced in 3 out of 4 studies [4,12,15]. Growth in the *Rikenellaceae* after the therapy was also visible [4,13].

## 4. Discussion

The correlation between exclusive enteral nutrition and the induction of remission was noticed among patients suffering from Crohn’s disease. Certainly, EEN has an impact on the gut microbiome and stimulates changes, which contribute to the recovery and withdrawal of some clinical symptoms. Although the exact mechanism remains elusive, retreatment of the inflammation can be obtained.

An association between the time of remission and time of intervention was visible. The accelerated change in the microbiota composition was achieved even after 1–2 weeks of EEN [12,15,16]. Nevertheless, the very first modification of the GI microbiome was not unequivocal with remission. Even the prolongation of the time of intervention (up to 12-weeks) may not bring more rapid clinical improvement, as indicated by Kaakoush et al. [13], where the mean time of achieving the remission was markedly long (19 weeks). However, the positive response to the treatment could be attributed to the short duration of the disease. In most cases, patients were newly diagnosed [4,13,16] and the decrease in the concentrations of hs-CRP, as well as the FCP level below 250 μg/g, were indicative of the improvement of the patients’ state [19]. Ashton et al. [4] reported 2.5 time less concentration of FCP, which corresponds to the achieved remission. It should be highlighted that a wide range of the standard deviation, observed in the obtained results of single studies after therapy, suggests a large difference between individual patient response, as some could obtained a great reduction, whereas other failed to achieve the remission rates. Interestingly, Logan et al. [19] reported no significant difference between FCP levels among patients who achieved the remission and these that did not benefit from EEN. It was suggested that the greater change in FCP level can be observed in the middle of the EEN therapy, and therefore, FCP can be recognized as a potential marker in order to distinguish patients in remission from the ones who did not maintain one [19]. It must be mentioned that EEN also plays a role in managing the body mass in patients, which is especially important in pediatric population, as proper nutrition is crucial for growth and development. The mean body weight gain ranged between 4.7 [13] and 2.4 kg [19]. The body weight changes are related not only to nutritional support provided to CD patient’s but also to the pharmacological treatment itself. Kang et al. [20] observed the significant differences in BMI Z-score after 8 weeks of EEN and corticosteroids therapy. As Gerasimidis et al. [21] indicated, weight gain was due to changes in lean, not fat mass. The reduction in the disease activity index (CDAI or PCDAI) was an amelioration of the patient’s clinical state; however, the state of remission can be defined in slightly different manners (Cut off: PCDAI< 10 or PCDAI < 15) [13,17]. This fact can cause an inconsistency while comparing achieved results. Interestingly, changes induced by the EEN retreat after the end of the therapy [8], which may suggest that EEN has a short term effect and no application as a maintenance therapy.

Besides positive outcomes of the EEN therapy, a few drawbacks can be mentioned, which influence the process of treatment. The unpalpable formula of the Modulen IBD, as well as the unpleasant smell and taste of the formula, makes patients draw back from the course of therapy [22]. This may also lead to the failure of the regimen. While the abundance of the bacteria in the GI is decreased after therapy, the data suggests that may be that the quantity of the species is not a decisive factor of the remission. Therefore, the proportion between the species can be an important factor. When the overall number of species lowers or does not change significantly, the abundance of individual groups can vary, with the dominance of the anti-inflammatory mediators producing species [15]. For example, the *Firmicutes* phylum includes 6 basic families and the remission does not need to be related to the relative lower abundance only but also to the different changes in proportion among them [13]. On the contrary, Shwerd et al. [15] reported the shift in relations between the families but no significant changes in the number of the OTU’s. It is worth mentioning that the *Firmicutes/Bacteroides* ratio is considered as a marker of the GI microbiome state. In healthy adults it oscillates around 10.9 and for elderly decreased up to 0.6 [23]. In the presented studies the *Firmicutes/Bacteroides* ratio after the EEN therapy was described at the level 1.04, which is closer to the results presented by the dysbiotic patients.

Lower abundance of *Faecalibacterium prausnitzii* was associated with butyrate production, and therefore protection of the mucosa, during the inflammatory diseases [17]. Interestingly, during the EEN, abundance of this bacteria lowered noticeably [8,13,14,16,17]. However, Gerasimidis et al. [16] indicated that the Modulen IBD formula has no fermentable fiber, so *F.prausnitzii* cannot produce short chain fatty acids (SCFA’s), which could be the reason for their lower abundance after EEN treatment. This may need further research, as in analyzed patients, formula composition could also modulate the observed effect. The mechanism underlying the effect of EN on the remission stays elusive. Even though it is known that it lowers the quantity of butyrate-producing bacteria. This fact alone can be portrayed as a downside of EEN because it has been shown that butyrate has a beneficial influence on epithelial barrier function [24]. Nevertheless, the reduction in toxins and inflammatory mediators producing species seems to play a more crucial role and the renovation of the epithelial barrier is suggested to play a major role [13,17].

Higher abundance of: *Erysipelotrichaceae*, *Ruminococcaceae*, *Lachnospiraceae*, *Streptococcaceae*, *Veillonellaceae* and *Peptostreptococcaceae* after EEN was observed [4,8,14,15,16]. Interestingly, Quince et al. [8] indicate that *Lactococcus,* the member of the *Lachnospiraceae* family, was the only genus that accelerated during therapy among 34 other studied species. This fact is coherent with the findings that anaerobic family as they have an ability to produce butyrate and SCFAs, they diminish the inflammation and improve peristaltic movements. Moreover, *Lachnospiraceae* family have an impact on the carbohydrates metabolism by enhancing the production of the GLP-1 and GLP-2 (glucagon like peptide 1 and 2), which boosts the insulin sensitivity [25]. It was also indicated by Leach et al. [26] that the positive correlation between the presence of *Prevotellaceae* family and disease activity index exists. It should be highlighted that the bacterial composition resembles 40% in structure from before the treatment [12], which suggests that EEN could be used as an instant intervention and bring the outcomes more rapidly but does not work as a permanent solution.

In all studies, genetic and innovating tools to assess the microbiome structure from faecal samples were used, which makes the comparison of their outcomes reliable. The main disadvantage was the heterogeneity in the manner of presenting the data. Authors used different formulas, like OTU’s, mean abundance, linear regression, or reads per sample, which implements inaccuracy in the mathematical and statistical comparison of the results. Furthermore, various taxonomy levels on which the studies were performed brings the deception to the interpretation of the results. However, it seems that the microbiome profiles assessment could be beneficial in prognosing response to the EEN therapy, which means that patients, who have the greatest chance to benefit from the treatment, may be specified even before start of the EEN [27].

## 5. Conclusions

In conclusion, an EEN influences positively on the GI microbiome structure, which seems to be interrelated with an alleviation of the symptoms and decrease in the process of inflammation and thus with the clinical remission of CD. EEN may modulate the relation between the GI bacterium families, which may lead to the total change in their abundance. Further studies are needed to give more evidence regarding EEN and microbiome.

## Figures and Tables

**Table 1:** Characteristic of included studies.

Study	Year	Country	Study Design	Study Population	Intervention	Time of Intervention (Weeks)
Ashton et al. [4]	2017	UK	NCT	CD = 3	EEN	6
				HS = 3	Modulen; (Nestle, Switzerland), 493 kcal/100 g, protein 14%, carbohydrates 44%, fats 42%, casein, corn syrup, sugar, milk fat, MCT, corn oil	
Schwerd et al. [15]	2016	Germany	NCT	CD = 8	EEN	3–4
					Modulen IBD (Nestle, Vevey, Switzerland): 493 kcal/100 g, protein 14%, carbohydrates 44%, fats 42%, casein, corn syrup, sugar, milk fat, MCT, corn oil or Neocate (Nutricia, Erlangen, Germany) 493 kcal/100 g, fats 47%, carbohydrates 42%, proteins 11%	
Kaakoush et al. [13]	2015	Australia	NCT	CD = 2	EEN	8–12
				HS = 5	Osmolite (Abbott Laboratories; Cronulla, NSW, Australia), 100 kcal/100 mL, protein 4 g, carbohydrates 13.56 g, fat 3.4 g, fibre 0 g, a polymeric formula	
Lewis et al. [12]	2015	United States	NCT	CD = 38	EEN 90% of calories from a not specified dietary formula;	8
				HS = 26	PEN: 53% of calories from formula	
Shiga et al. [10]	2012	Japan	NCT	CD = 8	Elental^®^ (Ajinomoto Co. Inc., Tokyo, Japan), 35–40 kcal/kg/day EEN, 35–40 kcal/kg/day TPN, carbohydrates 79.3%, amino acids 17.6%, fats	6
				HS = 12		
Jia et al. [17]	2010	UK	NCT	CD = 20	EEN	2
				HS = 18	E 028 Extra, Scientific Hospital Supplies International, Liverpool, UK, 443 kcal/100 g, 59% carbohydrates, 12.5% proteins, 17.5% fats	
D’Argenio et al. [14]	2013	Italy	CS	CD = 1	EEN	8
					Alicalm formula, Nutricia Advanced Medical Nutrition, 450 kcal/100 g, proteins 15%, carbohydrates 58%, fats 17.5%	
Gerasimidis et al. [16]	2014	UK	NCT	CD = 15	EEN	8
				HS = 21	Modulen; Nestlé UK Ltd., York, United Kingdom (polymeric casein-based liquid feed)	
					500 kcal/100 g, carbohydrates 54%, proteins 17.5%, fats 23%	
Quince et al. [8]	2015	UK	NCT	CD = 23	EEN	8
				HS = 21	Modulen, Nestle, Vevey, Switzerland, 493 kcal/100 g, protein 14%, carbohydrates 44%, fats 42%	

CD—Crohn’s disease, EEN—Exclusive Enteral Nutrition, PEN—Partial Enteral Nutrition, HS—Healthy Subjects, NCT—nonrandomized controlled trial, CS—case study.

**Table 2:** Characteristics of the study population (*n* = 118).

Study		Age (Years) MEAN ± SD	Sex (% Male)	Nationality	Diagnosis	Disease Location Paris Classification	Antibiotics Use	Steroids Use	Other Medication	Operation	Duration of the Disease (Years) MEAN	PCDAI/CDAI MEAN ± SD		CRP (mg/dl) MEAN ±SD		FCP (ug/g)– MEAN ± SD		Remission (Weeks) MEAN ±SD
												Baseline	Intervention	Baseline	Intervention	Baseline	Intervention	
Ashton et al. [4]		13.8 ±2.5	67	British	CD	L3 100% L4 33%	N/A	N/A	N/A	N/A	Newly diagnosed	N/A	N/A	N/A	N/A	664.7	248	3.3 ± 2.3
Schwerd et al. [15]		13.5 ± 2.2	53	German	CD	L1 13.64% L2 4.55% L3 45.46% L4 36.36%	N/A	N/A	N/A	N/A	1.5	43.125	11.875	2.0 ± 2.5	0.6 ± 0.9	N/A	N/A	No
Kaakoush et al. [13]		8.5 ± 1.8	100	Australian	CD	L3 + L4 50% L3 50%	N/A	N/A	N/A	N/A	newly diagnosed	38.75 ± 5.3	5 ± 7.1	40.5 ± 55.9	1	N/A	N/A	19 ± 9.9 *
Lewis et al. [12]	CD	12.6	63	American Canadian Hispanic	CD	L1 91% L2 68% L3 95% L4 58%	23.7%	35.7%	mesalamine 49% thiopurine 12%	5%	0.1 (range: 0.0–1.1)	32.5 (range: 20–45)	N/A	N/A	N/A	599.1 ± 626.76	594.32 ± 622.74	2.5 ± 1.12
	CD^s^	13.9	54	American Canadian Hispanic	CD	L1 82% L2 24% L3 96% L4 57%			mesalamine 50% thiopurine 4%	11%	0.2 (range: 0.1–1.0)	30 (range: 21.25–40)	N/A	N/A	N/A			
Shiga et al. [10]		30 (range 15–47)	82	N/A	CD	L2 9% L3 91%	N/A	N/A	N/A	47%	0–22	CDAI > 220	87.5%: < 150	1.3 0.1–3.6 (range)	0.2 0–0.6 (range)	N/A	N/A	26 (87.5% patients) **
Jia et al. [17]		N/A	N/A	N/A	CD	N/A	N/A	N/A	N/A	N/A	N/A	N/A	N/A	28.6 ± 36	4.9 ± 4.5	N/A	N/A	26 ***
D’Argenio et al. [14]		14	100	Italian	CD	N/A	N/A	N/A	N/A	N/A	N/A	50	0	N/A	N/A	N/A	N/A	8
Gerasimidis et al. [16]		12.7 (median)	67	British	CD	L3 + L4 60% L2 + L4 20% L2 13% L1 7%	N/A	N/A	azathioprine 33% aminosalicylates 27%	N/A	newly diagnosed 73%	No	22.5 <15 80%	N/A	N/A	2225	1574	8 (80% patients) ***
Quince et al. [8]		6.9–14.7 (range)	56	British	CD	L2 13% L2 + L4 17% L3 13% L3 + L4 57%	N/A	N/A	aminosalicylates 17% azathioprine 13%	N/A	N/A	40	<10 62%	24	7	2267	1686	8 (62% patients) *

* remission defined as PCDAI < 10, ** remission was defined as CDAI < 150, *** remission was defined as PCDAI < 15, CD^s^—patients whose baseline microbiome diversity was far more different from the healthy subjects.

**Table 3:** Microbiome assessment and diversity.

Study	Type of Samples	Method	Shannon Diversity Index MEAN ± SD			Abundance		
			before	after	control group	before	after	control group
Ashton et al. [4]	faecal samples	high-throughput 16S rRNA gene sequencing	4.84	5.20	5.6	8780 (observed species)	9599 (observed species)	11 119 (observed species) (cohabiting sibling controls)
Schwerd et al. [15]	faecal samples	high-throughput 16S rRNA gene sequencing	8–50 (range during treatment)		8–15	271 OTUs	no significant difference	N/A
Kaakoush et al. [13]	faecal samples	high-throughput 16S rRNA gene sequencing	2.25 ± 0.24	N/A	2.75 ± 0.14	121 ± 33 OTUs	N/A	117 ± 12 OTUs
Lewis et al. [12]	faecal samples	sequenced using the Illumina HiSeq paired-end method	N/A	N/A	N/A	3.49 × 10^9^ total reads	3.47 × 10^9^ total reads	N/A
Shiga et al. [10]	faecal samples	Terminal restriction fragment length polymorphism analysis of bacterial 16srDNA, Specific quantitative PCR	N/A	N/A	N/A	10.9 ± 0.6 (log10 cells per gram of faeces)	10.8 ± 0.8 (log10 cells per gram of faeces)	11.8 (log10 cells per gram of faeces)
Jia et al. [17]	faecal samples	PCR amplification, then intensity of the bands was measured and quantified by comparison with known amounts of a 1-kb ladder	N/A	N/A	N/A	Faecalibacterium prausnitzii A2-165 subgroup: 103 (average yield ng of PCR product generated)	Faecalibacterium prausnitzii A2-165 subgroup: 44 (average yield ng of PCR product generated)	170 (average yield ng of PCR product generated)
	blood samples		N/A	N/A	N/A	Faecalibacterium prausnitzii M21/2 subgroup: 127 (average yield ng of PCR product generated)	Faecalibacterium prausnitzii M21/2 subgroup: 113 (average yield ng of PCR product generated)	248 (average yield ng of PCR product generated)
D’Argenio et al. [14]	ileum tissue sample	16S rRNA next-generation sequencing strategy	3.9	6.2	7.1	705 OTUs	1328 OTUs	2171 OTUs
Gerasimidis et al. [16]	faecal samples	Quantitative Real-Time PCR	N/A	N/A	N/A	N/A	9 (median number of bands *)	11 (median number of bands)
Quince et al. [8]	faecal samples	16S rRNA gene sequencing and Shotgun metagenome sequencing	18.49	N/A	14.30	N/A	N/A	N/A

* bacterial diversity richness.

**Table 4:** Changes of microbiome—before and after treatment [%].

Study	Beneficial Bacteria		Pathogenic Bacteria	
	Increase	Decrease	Increase	Decrease
**Ashton et al. [4]**	Bacteroidaceae—402.80 Clostridiaceae—231.33 Coriobacteriaceae—162.76 Desulfovibrionaceae—115.26 Eubacteriaceae—19355.20 Rikenellaceae—596.53	Bifidobacteriaceae—42.79 Prevotellaceae—99.75	Erysipelotrichaceae—148.30 Lachnospiraceae—224.47 Porphyromonadaceae—94.32 Ruminococcae—58.06	Enterococcaceae—70.45 Pasteurellaceae—92.21 Enterobacteriaceae—10.58 Veilonellaceae—42.05
Schwerd et al. [15]	Coriobacteriaceae—47.87 Eubacteriaceae—118.18	Bacteroidaceae—77.92 Bifidobacteriaceae—99.82 Clostridiaceae—67.80 Desulfovibrionaceae—96.50 Prevotellaceae—95.54 Rikenellaceae—48.27	Erysipelotrichaceae—742.87 Lachnospiraceae—48.46	Enterobacteriaceae—14.15 Enterococcaceae—31.70 Porphyromonadaceae—66.99 Ruminococcae—76.64 Veilonellaceae—77.64
Kaakoush et al. [13]	Bacteroidaceae—49.40 Bifidobacteriaceae—8964.29 Desulfovibrionaceae—810.53 Rikenellaceae—5770.83	Clostridiaceae—42.26 Coriobacteriaceae—50.00 Prevotellaceae—100.00		Enterobacteriaceae–639.24 Enterococcaceae—68.29 Erysipelotrichaceae—36.84 Lachnospiraceae—53.74 Porphyromonadaceae—11.97 Ruminococcae—82.94
Lewis et al. [12]	Bifidobacteriaceae—4.86 Clostridiaceae—107.20 Coriobacteriaceae—653.70 Desulfovibrionaceae—15930.88 Rikenellaceae—169.96	Bacteroidaceae—5.59 Eubacteriaceae—30.03 Prevotellaceae—96.19	Enterococcaceae—9650.87 Erysipelotrichaceae—2082.34	Porphyromonadaceae—96.39
Shiga et al. [10]		Bacteroidaceae—94.00 Clostridiaceae–68.00		
Jia et al. [17]				Ruminococcaceae–32.00
D’Argenio et al. [14]	Bacteroidaceae–3468.12		Lachnospiraceae—33.41	Enterococcaceae–65.32
Gerasimidis et al. [16]		Bifidobacteriaceae–60.19	Lachnospiraceae–25.89	Enterococcaceae–60.19 Ruminococcaceae–90.00
Quince et al. [8]	Clostridiaceae–0.17		Lachnospiraceae—851.96	Ruminococcaceae—24.74
